# Impact of age and comorbidities on short- and long-term outcomes of patients undergoing surgery for colorectal cancer

**DOI:** 10.3389/fonc.2022.959650

**Published:** 2022-10-21

**Authors:** Giulia Turri, Gulser Caliskan, Cristian Conti, Luigi Martinelli, Ernesto De Giulio, Andrea Ruzzenente, Alfredo Guglielmi, Giuseppe Verlato, Corrado Pedrazzani

**Affiliations:** ^1^ Division of General and Hepatobiliary Surgery, Department of Surgical Sciences, Dentistry, Gynecology and Pediatrics, University of Verona, Verona, Italy; ^2^ Department of Diagnostic and Public Health, University of Verona, Verona, Italy

**Keywords:** elderly patients, colorectal cancer surgery, survival, comorbidities, mortality

## Abstract

**Background:**

As the world population is progressively ageing, more and more elderly patients will require cancer surgery. Although curative surgery is the treatment of choice for resectable colorectal cancer (CRC), it is still debated whether elderly frail patients should undergo major cancer surgery due to the increased risk of postoperative and long-term mortality. The aim of this retrospective study was to evaluate the impact of age and comorbidities on postoperative mortality/morbidity and long-term outcomes, looking for potential age-related survival differences.

**Methods:**

A total of 1,482 patients operated for CRC at our institution between January 2005 and October 2020 were analysed. The independent effect of age and comorbidities on postoperative complications was assessed by a logistic model, while the effect on overall survival (OS) and cancer-related survival (CRS) was estimated by a Cox regression model.

**Results:**

The median age in the cohort was 67.8 years. Postoperative mortality was very low in the whole cohort (0.8%) and contained even in older age groups (3.2% in patients aged 80–84 years, 4% in the 85–90-year age group). The cumulative incidence of postoperative complications was doubled in patients with comorbidities (32.8% vs. 15.1%, p = 0.002). With regard to OS, as expected, it exponentially decreased with advancing age. Conversely, differences in CRS were less pronounced between age groups and absent in patients with stage 0–I CRC. Analysis of all causes and cancer-related mortality revealed a peak within 2 years from surgery, suggesting a prolonged impact of surgery. In patients aged 75 years and above, all-cause mortality showed a steep increase 1 year after surgery, while cancer-related mortality plateaued at about 4 years after surgery. On multivariable analysis, OS, but not CRS, was significantly influenced by age.

**Conclusions:**

Although acceptable results of surgery in elderly patients, OS is strongly dependent on age: older people die more from competing causes than cancer-related treatments compared to younger age classes. The preoperative identification of risk factors for low OS may help the selection of those elderly patients who would benefit from curative CRC surgery.

## Introduction

Colorectal cancer (CRC) is one of the most common tumours worldwide, and its incidence increases with age, with a median age at diagnosis of 67 years (1). As the world population is progressively ageing, more and more elderly patients with CRC will require surgical treatment. Curative surgery is the treatment of choice for resectable CRC, and some current literature suggests that elderly patients have the same oncological benefit as younger patients (2, 3). However, it is still debated whether an invasive treatment should be performed in elderly patients (4, 5), due to the increased risk of postoperative complications, mortality, and difficulty to regain independence (6, 7). Furthermore, the definition of elderly is controversial. Even though the conventional definition of elderly refers to a person aged 65 years or more, frequently chronological age does not correspond to the biological one (8, 9). In fact, older age does not always correspond to frailty and more comorbidities. Therefore, to assess surgical risk in elderly patients, postoperative outcomes according to age classes were extensively investigated. Previous studies evaluated age-related morbidity and mortality in a short-term period, focusing on postoperative outcomes (10, 11). Despite the demonstration of acceptable short-term results after colorectal cancer surgery in older patients, the elderly population represents a heterogeneous cohort and may suffer from late complications as difficulty to thrive beyond 30 days after surgery (12, 13). Furthermore, 30-day mortality may underestimate surgical risk even in younger patients, as a not negligible proportion of them die beyond this time frame (14, 15). Interestingly, Dekker et al. showed a significant excess mortality in the first year after colorectal surgery in elderly patients, while those who survived thereafter showed the same cancer-related survival as younger patients (16). This excess mortality involved especially patients with comorbidities, higher stages of disease, emergency surgery, and postoperative complications, reaching 15%–30% in high-risk patients (17). Currently, the treatment of elderly patients with CRC represents a modern challenge of personalised medicine, balancing undertreatment based on the sole chronological age and overtreatment of frail patients (18–20).

The aim of this retrospective study was to evaluate the impact of age and comorbidities on postoperative mortality/morbidity and long-term outcomes on a large cohort of surgically treated CRC patients, and to evaluate the opportunity to submit elderly and frail patients to surgery.

## Material and methods

### Study population

**Figure 1:**
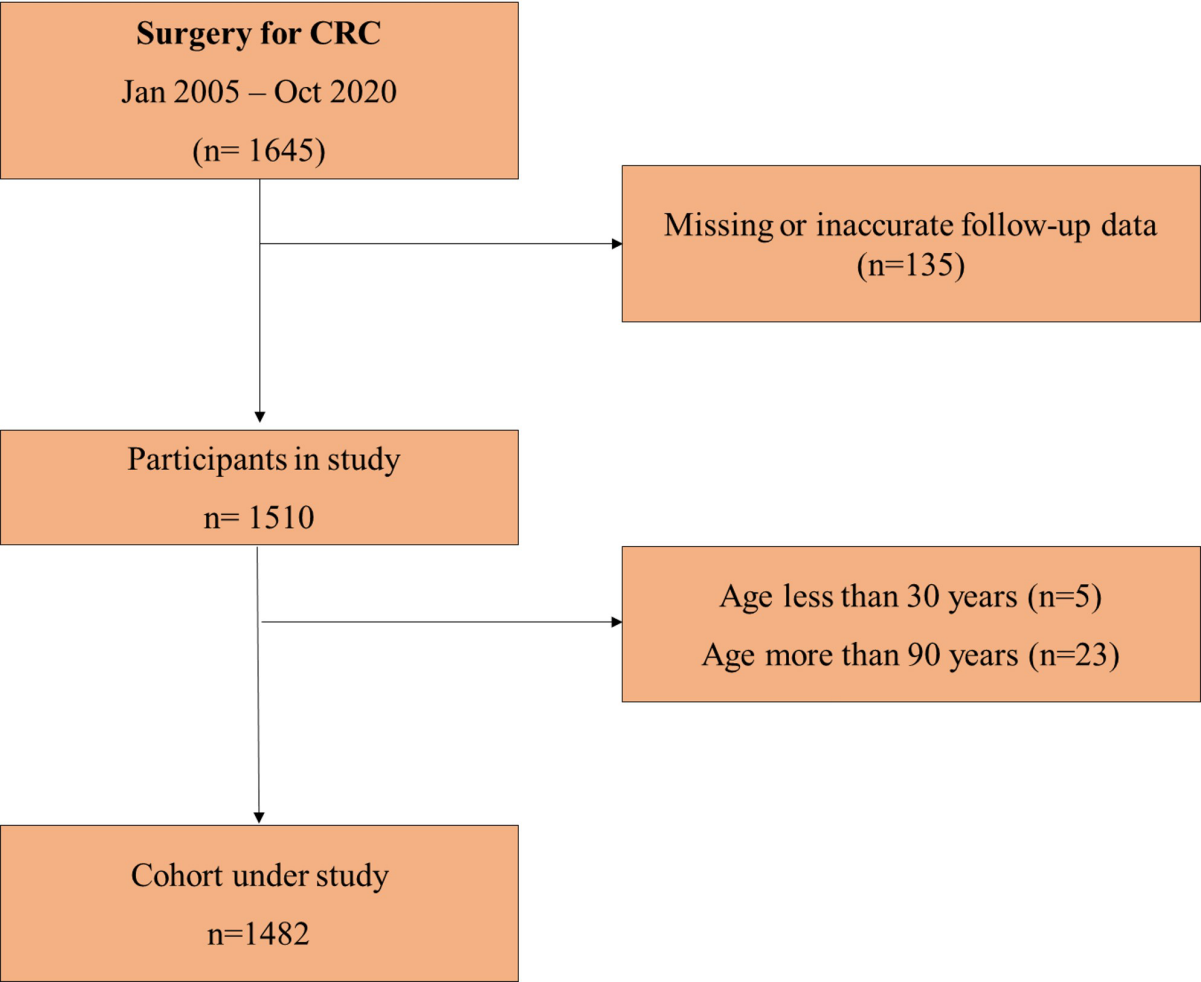
Flowchart depicting the selection process of patients under study.

The initial cohort of patients included 1,645 patients who had surgery for CRC at the Division of General and Hepatobiliary Surgery, University of Verona Hospital Trust, between January 2005 and October 2020. All elective and urgent surgeries and stage 0–IV, potentially curative (R0–1), and palliative (R2) procedures were included. Patients below the age of 30 and above the age of 90 were excluded, as the numbers in those age categories were very exiguous. Patients with missing follow-up data were also excluded. After application of inclusion criteria, 1,482 patients were analysed (**Figure 1**). Patients were classified in age classes as younger patients (<65 years) and elderly patients (65–69, 70–74, 75–79, 80–84, 85–89 years). Demographic, clinical, pathological, and pre- and postoperative data were retrieved from a retrospective database. All patients were staged with preoperative colonoscopy, chest–abdomen–pelvis computed tomography (CT), and carcinoembryonic antigen (CEA) measurement. The main goal of surgery was the complete excision of the cancer to obtain an R0 resection. The extent of the resection was planned according to cancer location, disease stage, and patient’s general conditions. Anatomical resections with ligation of vessels at their origin were the procedures of choice in order to achieve an adequate lymphadenectomy. The surgical approach included open and laparoscopic resections according to the surgeons’ preference, with laparoscopy becoming the preferred approach after 2014. Comorbidity status was assessed using the Charlson Age Comorbidity Index (CACI) (21). Tumours were staged according to the 8th Edition of the AJCC Cancer Staging Manual (22). Patients with locally advanced rectal cancer underwent long-course chemoradiotherapy or exclusive radiotherapy according to performance status and comorbidities. Preoperative chemotherapy was administered to patients with stage IV CRC depending on multidisciplinary assessment. Adjuvant chemotherapy was considered for patients with stage III/IV disease or stage II with risk factors after multidisciplinary discussion. Survival and follow-up data were obtained by revising outpatient clinical records for patients undergoing regular clinical follow-up or receiving oncological treatment at our centre. In the case of patients attending follow-up visits at other institutions, a member of our staff conducted a telephone follow-up at least once a year by directly contacting the patient or the relatives. Overall survival (OS) was defined as the length of time between primary surgery and time of death from any cause, whereas cancer-related survival (CRS) considered death from cancer or cancer-related treatment (i.e., postoperative mortality or toxicity/adverse events after chemotherapy) as the end point.

### Statistical analyses

The primary outcome variables were postoperative mortality and long-term OS and CRS. The main predictors considered in the present study were age, coded as <65, 65–69, 70–74, 75–79, 80–84, and 85–89 years, stage (stage 0–I, stage II, stage III, stage IV), gender (male, female), comorbidities (yes, no), tumour location (right colon, left colon, rectum), type of surgery (urgent, elective), neoadjuvant therapy (yes versus no), adjuvant therapy (yes versus no), radicality of surgery (R0–1 versus R2), postoperative complications (yes, no), and number of analysed lymph nodes (<12, ≥12). Significance of the association between age class and postoperative mortality was evaluated by Fisher’s exact test, and results are presented as n (%). Survival curves were estimated using the Kaplan–Meier method, and the log-rank test was used to evaluate the significance of differences among curves. To plot the estimated hazard function, a kernel smoother was used with a bandwidth of 0.2 years. The independent effect of age class on overall or cancer-related survival was evaluated by a Cox regression model, adjusting for gender, stage, comorbidity, and neoadjuvant and adjuvant chemotherapy. To test whether the prognostic significance of the main risk factors changed over time, the proportional hazards assumption of the Cox model was tested on the basis of Schoenfeld residuals. Life expectancy was compared between the CRC cohort and the general population from Verona. The comparison was restricted to patients undergoing R0–R1 surgery and to the age class 80–84 years, where median and mean survival could be computed for the CRC cohort. Life expectancy of the CRC cohort was extrapolated, as some patients were still alive at the end of follow-up, while life expectancy in the Verona general population was yielded by the Italian National Institute of Statistics (https://demo.istat.it/tvm2016). *p*-values below 0.05 were considered statistically significant. The statistical analysis was performed using STATA software, release 17.0 (StataCorp, College Station, TX, USA).

## Results

### Cohort under study

**Table 1** reports the main demographic and clinical characteristics of the included patients. The median age in the cohort was 67.8 years (IQR 58.8–76.9 years). The proportion of female patients increased significantly with increasing age (p < 0.001), as well as the median CACI (p < 0.001) and the presence of comorbidities (46.7% in patients aged below 65 years versus 77.6% in patients 85–89 years, p > 0.001). The percentage of patients undergoing surgery for rectal cancer decreased with increasing age (32.3% in patients aged <65 versus 10.5% in patients aged 85–89, p < 0.001), while the predominant site in the elderly was the right colon. Neoadjuvant treatment was more frequently used in younger patients (23.8% in patients aged <65 versus 7.9% in patients ages 80–84, p < 0.001). Interestingly, neoadjuvant treatment in patients aged <75 included both preoperative chemotherapy for metastatic CRC and neoadjuvant chemoradiotherapy for rectal cancer. On the contrary, most of patients aged >75 received neoadjuvant chemoradiotherapy for rectal cancer (p = 0.009).

**Table 1:** Characteristics of the cohort under study by age classes.

	Age classes						P value
	<65 years (n = 615)	65–69 years (n = 242)	70–74 years (n = 200)	75–79 years (n = 193)	80–84 years (n = 156)	85–89 years (n = 76)
**Gender, female**	279 (45.4%)	87 (36.0%)	69 (34.5%)	87 (45.1%)	75 (48.1%)	47 (61.8%)	**< 0.001**
**Comorbidities, yes**	287 (46.7%)	158 (65.3%)	140 (7.0%)	146 (75.6%)	138 (88.5%)	59 (77.6%)	**< 0.001**
**CACI, median (IQR)**	4 (3-5)	5 (4-6)	6 (5-7)	6 (5-7)	7 (6-8)	7 (6-8)	**< 0.001**
**CACI ≥5**	160 (25.8%)	109 (45%)	172 (86%)	166 (86.1%)	152 (96.8%)	65 (85.5%)	**< 0.001**
**BMI, median (IQR)**	24.8 (22.5-27.9)	25.5 (23.4-27.5)	25.6 (23.8-27.9)	24.9 (22.5-28.0)	26.1 (23.3-28.3)	24.2 (21.6-27.5)	0. 23
**Elective surgery**	593 (96.4%)	231 (95.5%)	194 (97.0%)	179 (92.8%)	143 (91.7%)	65 (85.5%)	**< 0.001**
**Laparoscopic surgery**	206 (33.5%)	83 (34.3%)	51 (25.5%)	47 (24.4%)	34 (21.8%)	22 (29.0%)	**0.008**
**Postoperative mortality**	0 (0.0%)	2 (0.8%)	0 (0.0%)	2 (1.0%)	5 (3.2%)	3 (4.0%)	**< 0.001**
**Postoperative complications**	139 (22.6%)	61 (25.2%)	53 (26.5%)	60 (31.1%)	54 (34.6%)	24 (31.6%)	**0.008**
Neoadjuvant therapy, yes*	129 (23.8%)	34 (16.0%)	18 (10.0%)	19 (11.2%)	11 (7.9%)	0 (0.0%)	**< 0.001**
**Neoadjuvant type** **CHT** **CRT** **RT**	53 (41.1%) 73 (56.6%) 3 (2.3%)	15 (44.1%) 17 (50.0%) 2 (5.9%)	9 (50.0%) 9 (50.0%) 0 (0.0%)	5 (26.3%) 13 (68.4%) 1 (5.3%)	1 (9.1%) 6 (54.5%) 4 (36.4%)	- - -	**0.009**
**Tumour location** **Left colon** **Right colon** **Rectum**	256 (41.6%) 165 (26.8%) 199 (32.3%)	95 (39.2%) 84 (34.7%) 63 (26.0%)	91 (45.5%) 66 (33.0%) 43 (21.5%)	68 (35.2%) 74 (38.3%) 51 (26.4%)	54 (34.6%) 64 (41.0%) 39 (25.0%)	27 (35.5%) 41 (53.9%) 8 (10.5%)	**< 0.001**
**Stage** **0–I** **II** **III** **IV**	197 (31.8%) 127 (20.5%) 151 (24.4%) 144 (23.3%)	84(34.7%) 62 (25.6%) 50 (20.7%) 46 (19%)	50 (25%) 64 (32%) 55 (27.5%) 31 (15.5%)	50 (26%) 67 (35%) 43 (22.4%) 32 (16.6%)	34 (21.8%) 55 (35.2%) 42 (27%) 25 (16%)	11 (14.5%) 29 (38.2%) 21 (27.6%) 13 (17.1%)	**< 0.001**
**Potentially curative (R0–1)**	539 (87.6%)	219 (90.5%)	176 (88%)	170 (88.1%)	140 (89.7%)	63 (82.9%)	0.540
**Adjuvant therapy, yes°**	93 (20.2%)	27 (14.5%)	21 (13.5%)	9 (6.0%)	6 (5.1%)	2 (3.8%)	**< 0.001**
**Number of retrieved lymph-nodes ≥12**	526 (87.1%)	204 (85.7%)	158 (81.0%)	148 (79.1%)	125 (81.2%)	54 (77.1%)	**0.027**

Data are expressed as number of patients (%) or median (IQR).

CACI (Charlson-Age Comorbidity Index); BMI (body max index).

*Information available for 1,317/1,482 patients.

°Information available for 1,121/1,482 patients. p values – 0.05 were highlighted in bold.

Most patients underwent elective surgery, but the proportion of urgent surgeries increased with increasing age (p < 0.001). Thirty-day postoperative mortality in the whole cohort was low and within acceptable ranges in all age groups (0.8%). Patients aged 80 years and above presented the highest postoperative mortality (3.2% in patients aged 80–84 years and 4% in patients aged 85–89 years, p < 0.001). Only patients with at least one comorbidity died in the postoperative period (12/928 = 1.3%). Finally, postoperative complications occurred more often in elderly patients (22.6% in patients aged below 65 versus 31.6% in patients aged 85–89 years, p = 0.008), and its occurrence was strictly associated with the presence of comorbidities (32.8% in patients with comorbidities versus 15.1% in patients without comorbidities, p = 0.002). Similarly to neoadjuvant treatment, adjuvant therapy was most frequently adopted in younger patients (20.2% in patients aged <65 versus 3.8% in patients aged 85–89, p < 0.001).

There was a non-linear relationship between stage and age. Stage 0–I and IV were more frequent in the youngest age group, where older patients presented a higher proportion of stage II and III CRC (p < 0.001). The radicality of surgery did not differ significantly between age groups as demonstrated by the similar proportions of potentially curative resections (R0–1).

### Long-term outcomes

As expected, OS markedly differed among the six age classes (**Supplementary Figure 1**, p <0.001) and decreased progressively with increasing age. The difference was blunted when considering only deaths related to cancer or cancer treatment (**Supplementary Figure 2**, p < 0.001). **Supplementary Tables 1–3** report OS and CRS stratified by age classes and gender, stages, and comorbidity status. OS was significantly poorer in older patients regardless of gender, stage, and comorbidities. With regard to CRS, male patients showed similar survival rates in all age classes (p = 0.198). When stratifying for stage, CRS did not differ significantly between the six age groups in patients with stage 0–I CRC (p = 0.072), but it was affected by age in the elderly groups. Finally, the presence of comorbidities influenced both OS (p < 0.001) and CRS (p = 0.012).

**Figure 2:**
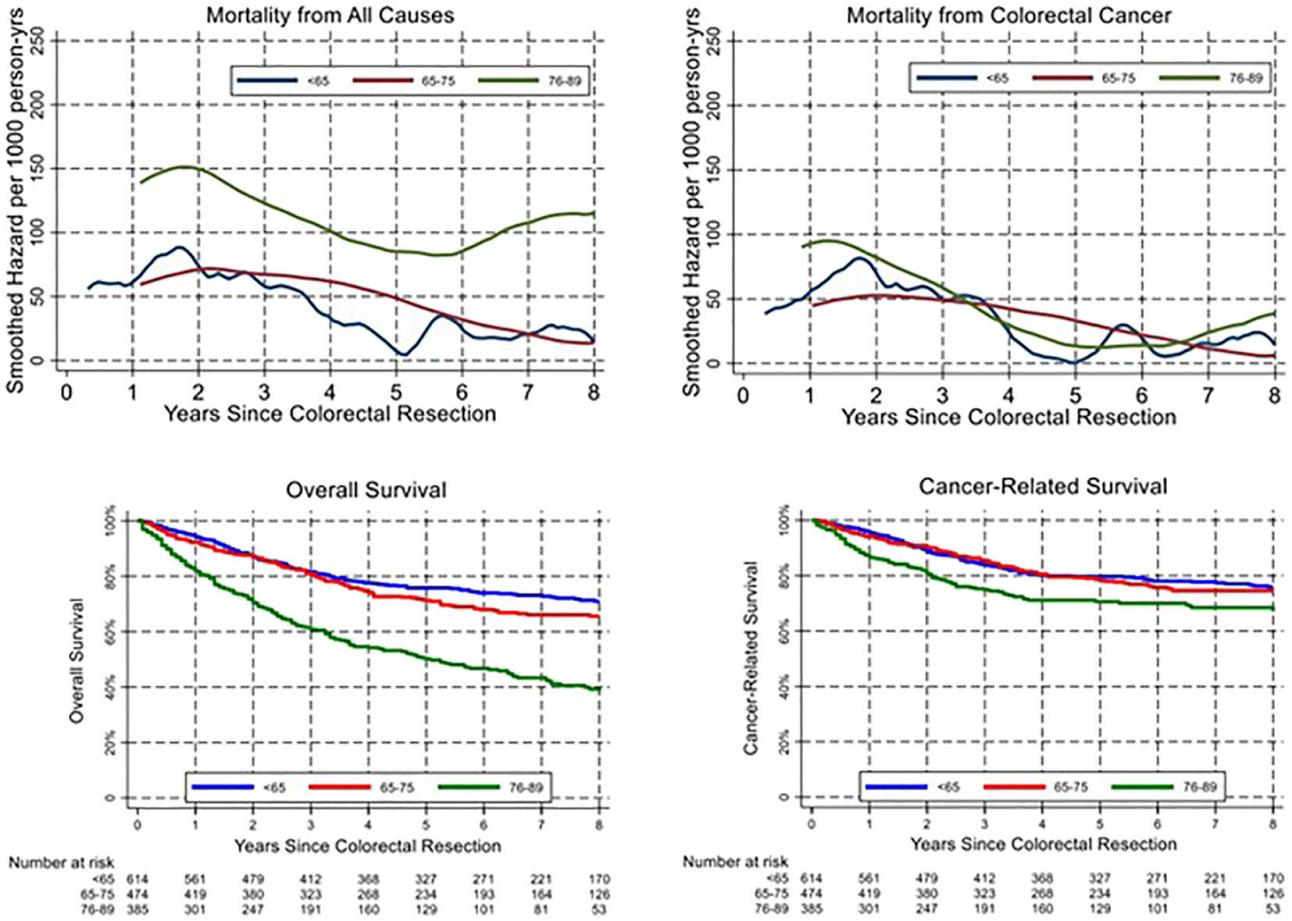
Temporal trend of all-cause and cancer-related mortality (upper panels) and corresponding overall and cancer-related survival (lower panels).

**Figure 3:**
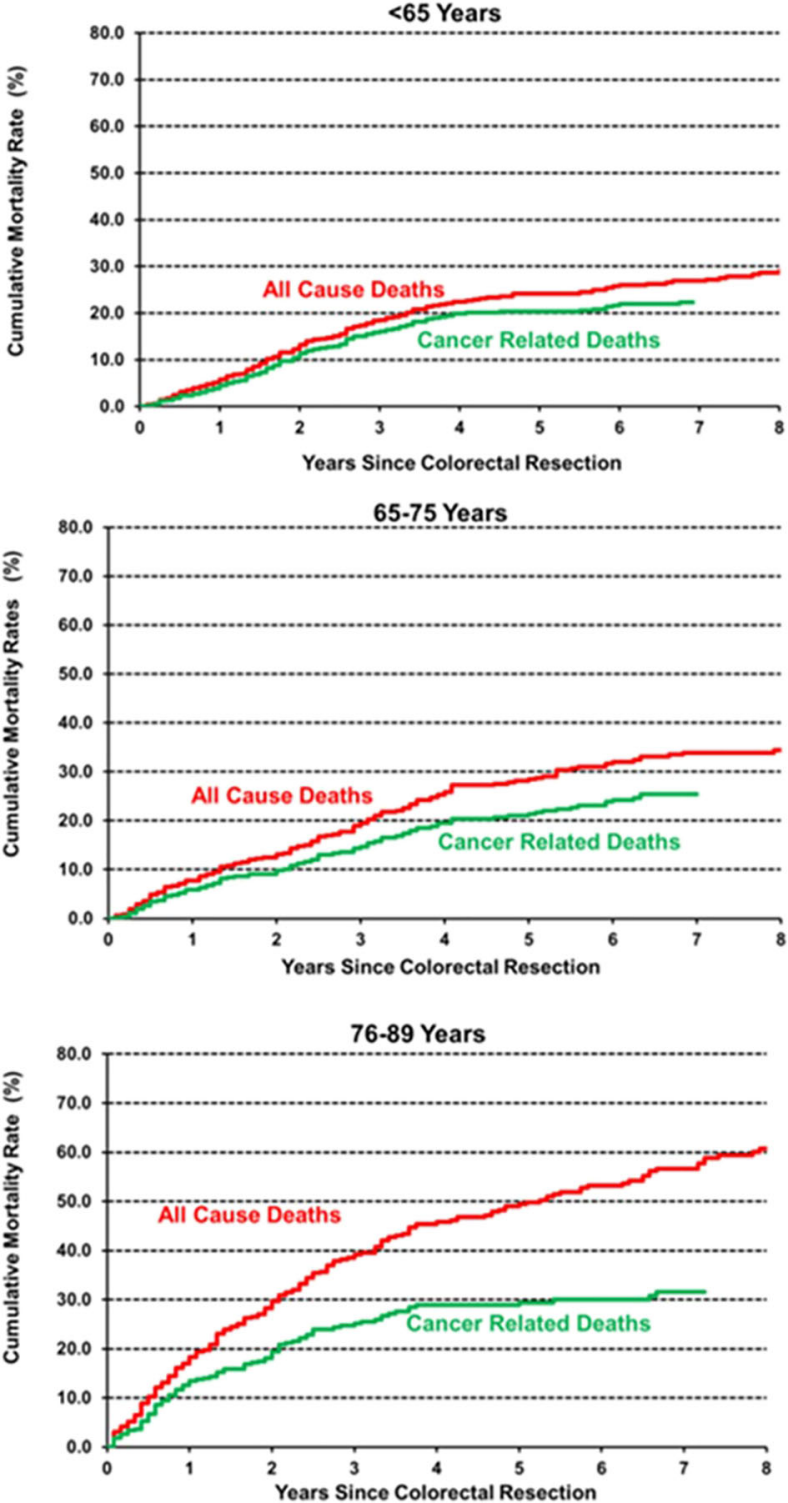
Cumulative incidence of all-cause and cancer-related mortality, estimated by the Kaplan–Meier method.

Age classes were then grouped into three categories to obtain adequate precision in estimating the hazard of mortality during follow-up: <65 years, 65–75, and 76–89 years. The smoothed hazard of mortality from CRC and from all causes is presented in the upper panel of **Figure 2**, while the lower panel shows survival curves estimated with the Kaplan–Meier method. Interestingly, the smoothed hazard of mortality from all causes was significantly higher in the 76–89 age class, while the other two age groups presented a similar hazard. The trend however was similar in all groups, with a peak of the hazard of all-cause mortality approximately 2 years after surgery and cancer-related mortality within the first 18 months. Cumulative cancer-related mortality and all-cause mortality were plotted separately for the three age groups using the Kaplan–Meier method (**Figure 3**). In younger patients, curves of cumulative all-cause mortality and cancer-related mortality over time were rather close throughout the follow-up time. On the contrary, the curves tended to separate already 1 year after surgery in CRC patients aged over 75 years, due to a larger mortality from causes other than cancer.

### Multivariable analysis

Multivariable analysis was conducted to assess the impact of age and comorbidities on OS and CRS (**Table 2**). OS was significantly influenced by age, comorbidities, stage, radicality of surgery, occurrence of postoperative complications, number of retrieved lymph nodes <12, and neoadjuvant treatment. On the contrary, age and comorbidities did not prove to be independent prognostic factors for CRS, which was only dependant on stage and neoadjuvant therapy.

**Table 2:** Multivariate analysis for overall and cancer-related survival.

	Overall survival		Cancer-related survival	
	Hazard ratio (95% CI)	P value	Hazard ratio (95% CI)	P value
**Age**				
<65	1 (reference)		1 (reference)	
65-69	0.95 (0.54-1.67)	0.87	0.86 (0.45-1.66)	0.66
70-74	1.75 (1.06- 2.89)	**0.03**	1.54 (0.84-2.82)	0.16
75-79	2.00 (1.21-3.33)	**0.007**	1.59 (0.83-3.01)	0.16
80-84	4.39 (2.70-7.16)	**< 0.001**	1.99 (0.97-4.08)	0.06
85-89	4.36 (2.28-8.33)	**< 0.001**	1.66 (0.56-4.98)	0.36
**Gender (male)**	1.03 (0.75-1.41)	0.83	0.96 (0.64-1.44)	0.84
**Tumour location**				
Left colon	1 (reference)		1 (reference)	
Right colon	0.95 (0.65-1.37)	0.76	1.04 (0.62-1.74)	0.87
Rectum	0.89 (0.52-1.51)	0.66	0.92 (0.47-1.79)	0.81
**Comorbidity (yes)**	1.72 (1.12-2.66)	**0.01**	1.33 (0.81-2.18)	0.25
**Type of surgery (urgent)**	1.00 (0.46-2.03)	0.95	0.69 (0.21-2.32)	0.55
**Postoperative complications (yes)**	1.49 (1.09-2.03)	**0.01**	1.44 (0.96-2.15)	0.07
**Stage**				
Stage 0-1	1 (reference)		1 (reference)	
Stage 2	2.25 (1.39-3.64)	**0.001**	2.98 (1.43-6.23)	**0.004**
Stage 3	5.09 (3.08-8.42)	**< 0.001**	9.26 (4.48-19.13)	**< 0.001**
Stage 4	8.06 (3.99-16.28)	**< 0.001**	13.14 (5.28-32.69)	**< 0.001**
**Potentially curative (R0-1)**	0.29 (0.16-0.54)	**< 0.001**	0.25 (0.13-0.49)	**< 0.001**
**Number of retrieved lymph-nodes <12**	1.73 (1.34-2.64)	**0.01**	1.71 (1.00-2.92)	**0.001**
**Neo-adjuvant therapy (yes)**	2.09 (1.18-3.69)	**0.01**	2.82 (1.43-5.56)	**0.003**
**Adjuvant therapy (yes)**	0.69 (0.43-1.10)	0.12	0.67 (0.39-1.15)	0.15

p values – 0.05 were highlighted in bold.

### Comparison between the CRC cohort and the general population

Life expectancy was compared between the CRC cohort, undergoing R0–R1 surgery, and the general population from Verona. The comparison was restricted to the age class 80–84 years, where the median and mean survival could be computed for the CRC cohort. Patients in stage 0–I had the same life expectancy of the Verona general population: life expectancy was 8.74 and 9.67 years, respectively, in male and female CRC patients, compared to 8.27 and 10.58 years in men and women from the Verona general population. Life expectancy was markedly reduced in stage III patients (5.3 years in men and 3.7 years in women) and furthermore in stage IV patients (2.3 and 2.2 years, respectively). Life expectancy in stage II patients was affected by gender, as it was similar to that of the general population in men (7.4 years) and substantially reduced in women (5.7 years).

## Discussion

The primary aim of the present study was to determine the long-term outcomes of elderly patients undergoing CRC surgery and to evaluate whether all age groups benefit from surgery. The treatment of CRC in elderly patients represents a contemporary dilemma as the world population is progressively ageing (23), and CRC exhibits a peak incidence around seventy years in both sexes (24).

Despite poorer OS in elderly patients, postoperative mortality was very low in the whole cohort (0.8%), and within acceptable ranges even in octogenarian patients (3%–4%). These data are in line with the current literature (25–27) and suggest that cancer surgery can be feasible with contained postoperative mortality even in older patients. It should be noted that postoperative mortality occurred only in patients aged 65 and above and with concurrent comorbidities, while younger and fit patients did not suffer any postoperative death. Prehabilitation as part of the Enhanced Recovery After Surgery (ERAS) protocol could play a role in the optimization of elderly CRC patients, and it may contribute to better surgical results (28). As previously published by our group, ERAS protocol can be safely applied to elderly patients undergoing laparoscopic colorectal resection with improvements in short-term postoperative outcomes (12).

In line with previous reports, we observed a higher proportion of advanced and early stages of CRC in younger patients (29). Conversely, elderly patients presented more often with stage II and stage III disease (p < 0.001). This finding may be due to a surgical selection bias, since more complex and aggressive treatments may have been offered to younger and healthier patients, while older patients with metastatic disease were directed towards palliative care. Similarly, less elderly patients underwent surgery for rectal cancer compared to the younger groups, whereas more elderly patients presented with right colon cancer (p < 0.001). This is in line with previous literature, which reports decreasing incidence of rectal cancer in patients aged >65 years (30).

In our cohort, OS differed significantly between age classes, as expected. On multivariable analysis, age remained a statistically relevant risk factor for OS, together with stage, presence of comorbidities, occurrence of postoperative complications, non-curative resection, inadequate lymphadenectomy, and neoadjuvant treatment. On the other hand, whereas CRS was lower in older patients (**Supplementary Figure 2**), age did not prove to be an independent prognostic factor on multivariable analysis. Interestingly, neoadjuvant treatment emerged as an independent negative prognostic factor for OS and CRS on multivariable analysis. This result may be explained by the association between neoadjuvant treatment and a more advanced disease at diagnosis (locally advanced rectal cancer or stage IV CRC). When analysing the trend in overall and cancer-related mortality, we observed a peak within 2 years from surgery in all age groups and more pronounced in elderly patients (**Figure 2**). These results are only partly in line with previous literature that identified an excess mortality at the first year after surgery (17). On the contrary, our results suggest a prolonged impact of surgery beyond the first year and the peak in mortality within 18 and 24 months after cancer surgery. As shown in **Figure 3** with Kaplan–Meier estimates of all-cause and cancer-related deaths, younger patients with CRC die almost always due to cancer progression or treatment-related complications. Conversely, the curves for elderly patients diverge quite steeply right after the first year, suggesting more deaths from competing causes.

Interestingly, life expectancy at 80–84 years of age was similar between stage 0–I CRC patients and the general population of Verona (8.74 vs. 8.27 years, respectively, for men, 9.67 vs. 10.58 years for women), suggesting a low impact of surgical treatment. However, life expectancy of stage III and stage IV CRC was markedly reduced in both sexes. These data suggest that curative surgery can be safely performed also in elderly patients with CRC, with important benefits on OS if they do not die of competing causes and they present with resectable and early-stage disease. Palliative surgery or extensive resections for stage IV CRC, however, do not provide survival benefits.

From the results of our study and from current literature, we could conclude that elderly CRC patients should not be undertreated just because of their chronological age. A careful preoperative evaluation should select elderly fit patients for low-risk surgery. Frail and comorbid patients should otherwise be directed towards medical optimization and prehabilitation before surgery (31–33). The definition of frail patient is not unique: frailty may be defined as “a state of decreased physiologic reserve caused by the accumulation of ageing processes across multiple organ systems, which affects the patient’s resistance to stressors” (34). Different tools for the assessment of frailty have been proposed, but they are often too time consuming to be routinely used in clinical practice (35, 36). Montroni et al. have recently proposed other more immediate tools to identify frail patients undergoing general surgery with a particular emphasis to assess the quality of life and the functional recovery after cancer surgery too (37, 38). However, all these scores focus on the identification of patients at risk for postoperative complications and short-term mortality, while they do not consider correlation with long-term mortality. Further studies are required to better define who could benefit from surgery and who should be spared because they are too frail and at risk of early mortality.

Despite including a large number of patients with a long follow-up time, our study presents some limitations. Due to its retrospective nature, it was not possible to retrieve complete data on some variables, including the administration of neoadjuvant/adjuvant therapy and the complications related to perioperative oncological treatment. Also, retrieval of the cause of the death was sometimes limited by the possibility to directly contact the patients or their relatives.

In conclusion, CRC surgery may be offered even to elderly patients with acceptable postoperative mortality. However, it should be considered that there is a more pronounced increase in 2-year all-cause mortality in elderly patients, suggesting a prolonged impact of surgery.

## Data availability statement

The dataset will be available on reasonable request at the corresponding author. Requests to access the datasets should be directed to corrado.pedrazzani@univr.it.

## Ethics statement

All methods used in this study were performed in accordance with the relevant ethical guidelines and regulations of the University Hospital of Verona, where the investigation was carried out. Informed consent was obtained from all patients and the study protocol was approved by the local ethical committee (ID: 1560 CESC).

## Author contributions

GT, GC, CC, GV, and CP contributed to conception and design of the study. GT, GC, and LM organized the database. GC, LM, and GV performed the statistical analysis. GT, GC, CC, LM, EG, CP, and GV wrote the first draft of the manuscript. AR, AG, GV, and CP contributed to interpretation of the results and critical revision. All authors contributed to manuscript revision, read, and approved the submitted version.

## Conflict of interest

The authors declare that the research was conducted in the absence of any commercial or financial relationships that could be construed as a potential conflict of interest.

## Publisher’s note

All claims expressed in this article are solely those of the authors and do not necessarily represent those of their affiliated organizations, or those of the publisher, the editors and the reviewers. Any product that may be evaluated in this article, or claim that may be made by its manufacturer, is not guaranteed or endorsed by the publisher.

## References

[1] Howlader N Noone AM Krapcho M Miller D Bishop K Kosary CL. SEER cancer statistics review, 1975-2016. (Bethesda, MD: National Cancer Institute) (2019).

[2] Jung B Påhlman L Johansson R Nilsson E. Rectal cancer treatment and outcome in the elderly: An audit based on the Swedish rectal cancer registry 1995-2004. BMC Cancer (2009) 9:68. doi: 10.1186/1471-2407-9-68 19245701 PMC2653041

[3] Samuelsson KS Egenvall M Klarin I Lökk J Gunnarsson U. Preoperative geriatric assessment and follow-up of patients older than 75 years undergoing elective surgery for suspected colorectal cancer. J Geriatric Oncol (2019) 10(5):709–15. doi: 10.1016/j.jgo.2019.01.020Al-Refaie 30745117

[4] Al-Refaie WB Parsons HM Habermann EB Kwaan M Spencer MP Henderson WG. Operative outcomes beyond 30-day mortality: colorectal cancer surgery in oldest old. Ann Surg (2011) 253(5):947–52. doi: 10.1097/SLA.0b013e318216f56e 21490452

[5] Chang GJ Skibber JM Feig BW Rodriguez-Bigas M. Are we undertreating rectal cancer in the elderly? an epidemiologic study. Ann Surg (2007) 246(2):215–21. doi: 10.1097/SLA.0b013e318070838f PMC1933551 17667499

[6] Janssen-Heijnen MLG Maas HAAM Saskia Houterman Lemmens VEPP Rutten HJT Coebergh JWW. Comorbidity in older surgical cancer patients: influence on patient care and outcome. Eur J Cancer (Oxford Engl 1990) (2007) 43(15):2179–93. doi: 10.1016/j.ejca.2007.06.008 17681780

[7] Montroni I Rostoft S Spinelli A Van Leeuwen BL Ercolani G Saur NM. GOSAFE - Geriatric oncology surgical assessment and functional rEcovery after surgery: early analysis on 977 patients. J Geriatric Oncol (2020) 11(2):244–55. doi: 10.1016/j.jgo.2019.06.017 31492572

[8] WHO. Definition of an older or elderly person (2010). Geneva: Switzerland World Health Organisation (Accessed 12/11/2013).

[9] Jazwinski SM Kim S. Examination of the dimensions of biological age. *Frontiers in genetics* . Front Genet (2019) 10:263. doi: 10.3389/fgene.2019.00263 30972107 PMC6445152

[10] Morris EJA Taylor EF Thomas JD Quirke P Finan PJ Coleman MP. Thirty-day postoperative mortality after colorectal cancer surgery in England. Gut (2011) 60(6):806–13. doi: 10.1136/gut.2010.232181 21486939

[11] Byrne BE Mamidanna R Vincent CA Faiz O. Population-based cohort study comparing 30- and 90-day institutional mortality rates after colorectal surgery. Br J Surg (2013) 100(13):1810–7. doi: 10.1002/bjs.9318 PMC4065361 24227369

[12] Pedrazzani C Conti C Turri G Lazzarini E Tripepi M Scotton G. Impact of age on feasibilty and short-term outcomes of ERAS after laparoscopic colorectal resection. World J Gastrointest Surg (2019) 11(10):395–406. doi: 10.5498/wjp.v2.i2.26 31681461 PMC6821935

[13] Gooiker GA Dekker JWT Bastiaannet E van der Geest LGM Merkus JWS van de Velde CJH. Risk factors for excess mortality in the first year after curative surgery for colorectal cancer. Ann Surg Oncol (2012) 19(8):2428–34. doi: 10.1245/s10434-012-2294-6 PMC3404283 22396000

[14] Visser BC Keegan H Martin M Wren SM. Death after colectomy: It’s later than we think. Arch Surg (2009) 144(11):1021–7. doi: 10.1001/archsurg.2009.197 19917938

[15] Mamidanna R Almoudaris AM Faiz O. Is 30-day mortality an appropriate measure of risk in elderly patients undergoing elective colorectal resection? Color Dis (2012) 14(10):1175–82. doi: 10.1111/j.1463-1318.2011.02859.x 21999306

[16] Dekker JWT van den Broek CBM Bastiaannet E van de Geest LGM Tollenaar RAEM Liefers GJ. Importance of the first postoperative year in the prognosis of elderly colorectal cancer patients. Ann Surg Oncol (2011) 18(6):1533–9. doi: 10.1245/s10434-011-1671-x PMC3087879 21445672

[17] Dekker JWT Gooiker GA Bastiaannet E van den Broek CBM van der Geest LGM van de Velde CJ. Cause of death the first year after curative colorectal cancer surgery; A prolonged impact of the surgery in elderly colorectal cancer patients”. Eur J Surg Oncol J Eur Soc Surg Oncol Br Assoc Surg Oncol (2014) 40(11):1481–7. doi: 10.1016/j.ejso.2014.05.010 24985723

[18] Janssen-Heijnen MLG Houterman S Lemmens VEPP Louwman MWK Coebergh JWW. Age and co-morbidity in cancer patients: A population-based approach. Cancer Treat Res (2005) 124:89–107. doi: 10.1007/0-387-23962-6_5 15839192

[19] Bojer AS Roikjær O. Elderly patients with colorectal cancer are oncologically undertreated. Eur J Surg Oncol (2015) 41(3):421–5. doi: 10.1016/j.ejso.2014.10.065 25592663

[20] Bahadoer RR Bastiaannet E Claassen YHM van der Mark M van Eycken E Verbeeck J. One-year excess mortality and treatment in surgically treated patients with colorectal cancer: A EURECCA European comparison. Eur J Surg Oncol J Eur Soc Surg Oncol Br Assoc Surg Oncol (2021) 47(7):1651–60. doi: 10.1016/j.ejso.2021.01.01 33518367

[21] Charlson ME Pompei P Ales KL MacKenzie CR. A new method of classifying prognostic comorbidity in longitudinal studies: Development and validation. J Chronic Dis (1987) 40(5):373–83. doi: 10.1016/0021-9681(87)90171-8 3558716

[22] Weiser MR. AJCC 8th edition: Colorectal cancer. Ann Surg Oncol (2018) 25(6):1454–5. doi: 10.1245/s10434-018-6462-1 29616422

[23] United Nations, Department of Economic and Social Affairs, Population Division. World population prospects. In: The 2017 revision, key findings and advance tables (2017) United Nations, New York.

[24] Available at: https://www.aiom.it/wp-content/uploads/2020/10/2020_Numeri_Cancro-operatori_web.pdf.

[25] Brenner H Bouvier AM Foschi R Hackl M Larsen IK Lemmens V. Progress in colorectal cancer survival in Europe from the late 1980s to the early 21st century: the EUROCARE study. Int J Cancer (2012) 131(7):1649–58. doi: 10.1002/ijc.26192 21607946

[26] Ketelaers SHJ Orsini RG Burger JWA Nieuwenhuijzen GAP Rutten HJT. Significant improvement in postoperative and 1-year mortality after colorectal cancer surgery in recent years. Eur J Surg Oncol J Eur Soc Surg Oncol Br Assoc Surg Oncol (2019) 45(11):2052–8. doi: 10.1016/j.ejso.2019.06.017 31255442

[27] Breugom AJ Bastiaannet E Dekker JWT Wouters MWJM van de Velde CJH Liefers GJ.. Decrease in 30-day and one-year mortality over time in patients aged ≥75 years with stage I-III colon cancer: A population-based study. Eur J Surg Oncol (2018) 44(12):1889–93. doi: 10.1016/j.ejso.2018.08.010 30262327

[28] Gustafsson UO Scott MJ Hubner M Nygren J Demartines N Francis N. Guidelines for perioperative care in elective colorectal surgery: Enhanced recovery after surgery (ERAS^®^) society recommendations: 2018. World J Surg (2019) 43(3):659–95. doi: 10.1007/s00268-018-4844-y 30426190

[29] Chou CL Chang SC Lin TZ Chen WS Jiang JK Wang HS. Differences in clinicopathological characteristics of colorectal cancer between younger and elderly patients: An analysis of 322 patients from a single institution. Am J Surg (2011) 202(5):574–82. doi: 10.1016/j.amjsurg.2010.10.014 21872205

[30] Siegel RL Fedewa SA Anderson WF Miller KD Ma J Rosenberg PS. Colorectal cancer incidence patterns in the united states, 1974-2013. J Natl Cancer Inst (2017) 109(8):djw322. doi: 10.1093/jnci/djw32 PMC6059239 28376186

[31] Morley JE Vellas B van Kan GA Anker SD Bauer JM Bernabei R. Frailty consensus: A call to action. J Am Med Directors Assoc (2013) 14(6):392–7. doi: 10.1016/j.jamda.2013.03.022 PMC4084863 23764209

[32] Gillis C Li C Lee L Awasthi R Augustin B Gamsa A. Prehabilitation versus rehabilitation: A randomized control trial in patients undergoing colorectal resection for cancer. Anesthesiology (2014) 121(5):937–47. doi: 10.1097/ALN.0000000000000393 25076007

[33] Carli F Minnella EM. Preoperative functional assessment and optimization in surgical patient: changing the paradigm. Minerva Anestesiol (2017) 83(2):214–8. doi: 10.23736/S0375-9393.16.11564-0 27711026

[34] Balducci L. Aging, frailty, and chemotherapy. Cancer Control (2007) 14(1):7–12. doi: 10.1177/107327480701400102 17242666

[35] Monfardini S Balducci L. A comprehensive geriatric assessment (CGA) is necessary for the study and the management of cancer in the elderly. Eur J Cancer (1999) 35(13):1771–2. doi: 10.1016/s0959-8049(99)00227-0 10673990

[36] Kristjansson SR Nesbakken A Jordhøy MS Skovlund E Audisio RA Johannessen HO. Comprehensive geriatric assessment can predict complications in elderly patients after elective surgery for colorectal cancer: A prospective observational cohort study. Crit Rev Oncology/Hematology (2010) 76(3):208–17. doi: 10.1016/j.critrevonc.2009.11.002 20005123

[37] Montroni I Ugolini G Saur NM Spinelli A Rostoft S Millan M. Personalized management of elderly patients with rectal cancer: Expert recommendations of the European society of surgical oncology, European society of coloproctology, international society of geriatric oncology, and American college of surgeons commission on cancer. Eur J Surg Oncol J Eur Soc Surg Oncol Br Assoc Surg Oncol (2018) 44(11):1685–702. doi: 10.1016/j.ejso.2018.08.003 30150158

[38] Montroni I Ugolini G Saur NM Rostoft S Spinelli S Van Leeuwen BL. Quality of life in older adults after major cancer surgery: The GOSAFE international study. J Natl Cancer Institute (2022) 114:djac071. doi: 10.1093/jnci/djac071 PMC9275771 35394037

