# Supplementary material for: Impact of age and comorbidities on short- and long-term outcomes of patients undergoing surgery for colorectal cancer
Source: Front Oncol. 2022 Oct 21;12:959650. doi: 10.3389/fonc.2022.959650 (PMC9633938; doi:10.3389/fonc.2022.959650)
Supplement: Supplementary file 1 [file DataSheet_1.docx]

**Supplementary Figure 1.** Overall survival in six different age classes (<65, 65-69, 70-74, 75-79, 80-84, 85-89 years), estimated by Kaplan-Meier method.

**Supplementary figure 2.** Cancer-related survival in six different age classes (<65, 65-69, 70-74, 75-79, 80-84, 85-89 years), estimated by Kaplan-Meier method.
