# Supplementary material for: Impact of age and comorbidities on short- and long-term outcomes of patients undergoing surgery for colorectal cancer
Source: Front Oncol. 2022 Oct 21;12:959650. doi: 10.3389/fonc.2022.959650 (PMC9633938; doi:10.3389/fonc.2022.959650)
Supplement: Supplementary file 2 [file DataSheet_2.docx]

**Supplementary Table 1**. OS and CRS by Gender and Age Group

|  | | **<65 years** | **65-69 years** | **70-74 years** | **75-79 years** | **80-84 years** | **85-89 years** | **P Value** |
| --- | --- | --- | --- | --- | --- | --- | --- | --- |
| **OS** | **Males** | 75.9%  (70.5-80.4%) | 72.4%  (63.9-79.3%) | 69.2%  (59.9-76.9%) | 59.7%  (49.0-68.8%) | 46.0%  (34.0-57.2%) | 54.7%  (28.9-74.5%) | **<0.001** |
|  | **Females** | 75.7%  (69.8-80.6%) | 80.2%  (69.2-87.7%) | 63.1%  (49.8 –73.8%) | 60.9%  (49.1–70.8%) | 46.8%  (33.9-58.7%) | 33.8%  (19.1-49.1%) | **<0.001** |
| **CRS** | **Males** | 79.7%  (74.6-83.9%) | 79.7%  (74.6-83.9%) | 77.3%  (68.3-84.0%) | 73.0%  (62.6-81.0%) | 70.4%  (57.6-79.9%) | 90.1%  (64.9-97.5%) | 0.198 |
|  | **Females** | 79.6%  (73.9-84.2%) | 85.9%  (75.1-92.3%) | 70.2%  (56.8-80.2%) | 71.3%  (59.5-80.2%) | 67.2%  (52.8-78.0%) | 66.3%  (48.7-79.1%) | **0.005** |

**Supplementary Table 2**. OS and CRS by Stage and Age Group

|  | | **<65 years** | **65-69 years** | **70-74 years** | **75-79 years** | **80-84 years** | **85-89 years** | **P Value** |
| --- | --- | --- | --- | --- | --- | --- | --- | --- |
| **OS** | **Stage 0-1** | 98.0%  (94.3-99.1%) | 91.6%  (82.2-96.2%) | 86.0%  (71.2-93.5%) | 86.3%  (72.0- 94.0%) | 65.9%  (44.4-81.0%) | 90.9%  (50.8-98.7%) | **<0.001** |
|  | **Stage 2** | 88.1%  (80.4-92.9%) | 85.5%  (71.8-92.9%) | 77.9%  (64.9-86.6%) | 69.4%  (56.2-79.3%) | 55.5%  (40.6-68.1%) | 46.9%  (25.9-65.4%) | **<0.001** |
|  | **Stage 3** | 76.8%  (68.3-83.3%) | 77.2%  (61.6-87.1%) | 67.1%  (51.7-78.5%) | 40.1%  (24.2-55.4%) | 41.4%  (25.1-56.9%) | 42.9%  (20.4-63.6%) | **<0.001** |
|  | **Stage 4** | 28.4%  (19.9-37.3%) | 25.7%  (11.7-42.4%) | 9.1%  (1.6-24.8%) | 21.1%  (7.5-39.3%) | 7.5%  (0.7-26.3%) | 0%  (0-0%) | **<0.001** |
| **CRS** | **Stage 0-1** | 98.3%  (94.9-99.5%) | 98.6%  (90.4-99.8%) | 94.8%  (80.5-98.7%) | 93.1%  (79.7-97.8%) | 91.5%  (69.2-97.9%) | 90.9%  (50.8-98.7%) | 0.072 |
|  | **Stage 2** | 92.8%  (86.1-96.3%) | 90.9%  (77.5-96.5%) | 88.9%  (76.8-94.9%) | 82.8%  (70.3-90.4%) | 78.5%  (63.5-87.9%) | 92.1%  (71.9-97.9%) | **0.017** |
|  | **Stage 3** | 79.8%  (71.5-85.9%) | 80.8%  (65.1-89.9%) | 73.8%  (58.9-83.9%) | 55.1%  (36.7-70.1%) | 63.2%  (42.3-78.3%) | 75.9%  (47.2-90.5%) | **0.026** |
|  | **Stage 4** | 34.6%  (25.1-44.2%) | 31.7%  (14.5-50.5%) | 10.0%  (1.8-26.8%) | 29.4%  (11.9-49.7.%) | 9.6%  (0.08-32.2%) | 0%  (0-0%) | **<0.001** |

**Supplementary Table 3**. OS and CRS by Comorbidity and Age Group

|  |  | **<65 years** | **65-69 years** | **70-74 years** | **75-79 years** | **80-84 years** | **85-89 years** | **P Value** |
| --- | --- | --- | --- | --- | --- | --- | --- | --- |
| **OS** | **Yes** | 74.9%  (68.9-80.0%) | 75.7%  (67.2-82.3%) | 62.4%  (52.9-70.6%) | 57.8%  (48.6-65.9%) | 44.3%  (35.1-53.2%) | 36.2%  (20.9-51.8%) | **<0.001** |
|  | **No** | 78.2%  (71.9-83.2%) | 77.5%  (62.9-86.9%) | 86.1%  (66.9-94.6%) | 75.9%  (47.7-90.2%) | 63.5%  (28.5-84.8%) | 55.6%  (7.3-87.6%) | **0.014** |
| **CRS** | **Yes** | 80.2%  (74.5-84.8%) | 84.3%  (76.2-89.8%) | 71.4%  (61.9-78.9%) | 72.6%  (63.6-79.7%) | 68.9%  (58.8-76.9%) | 73.9%  (57.3-84.8%) | **0.012** |
|  | **No** | 80.2%  (73.9-85.0%) | 79.6%  (65.2-88.5%) | 89.8%  (71.7-96.6%) | 75.9%  (47.7-90.2%) | 74.0%  (38.2-91.0%) | 83.3%  (27.3-97.5%) | 0.478 |
